# Immune Checkpoints as Therapeutic Targets in Autoimmunity

**DOI:** 10.3389/fimmu.2018.02306

**Published:** 2018-10-08

**Authors:** Christopher Paluch, Ana Mafalda Santos, Consuelo Anzilotti, Richard J. Cornall, Simon J. Davis

**Affiliations:** ^1^MRC Human Immunology Unit, University of Oxford, Oxford, United Kingdom; ^2^Nuffield Department of Clinical Medicine, University of Oxford, Oxford, United Kingdom; ^3^Radcliffe Department of Medicine, University of Oxford, Oxford, United Kingdom

**Keywords:** immune checkpoint, inhibitory receptor, agonist, antibody, autoimmunity, immunosuppression

## Abstract

Antibodies that block the immune checkpoint receptors PD1 and CTLA4 have revolutionized the treatment of melanoma and several other cancers, but in the process, a new class of drug side effect has emerged—immune related adverse events. The observation that therapeutic blockade of these inhibitory receptors is sufficient to break self-tolerance, highlights their crucial role in the physiological modulation of immune responses. Here, we discuss the rationale for targeting immune checkpoint receptors with agonistic agents in autoimmunity, to restore tolerance when it is lost. We review progress that has been made to date, using Fc-fusion proteins, monoclonal antibodies or other novel constructs to induce immunosuppressive signaling through these pathways. Finally, we explore potential mechanisms by which these receptors trigger and modulate immune cell function, and how understanding these processes might shape the design of more effective therapeutic agents in future.

## Introduction

### Immune checkpoint receptors

The immune system comprises a powerful arsenal of effector mechanisms capable of inflicting devastating damage on invading pathogens, but also with the capacity to do great harm to the body itself. In order to prevent such destruction of host tissues and to restore quiescence after an inflammatory response, careful immune regulation is required. In the periphery, immune cell responses are controlled by a balance between positive and negative signals, which attune effector cells to their environment. For a T cell these signals are delivered by a myriad of co-stimulatory and co-inhibitory surface receptors, whose inputs are integrated alongside T cell receptor (TCR) signaling to determine the cell's fate. The co-inhibitory receptors such as programmed cell death protein 1 (PD1) and cytotoxic T lymphocyte associated protein 4 (CTLA4), also known as immune checkpoints, recognize surface-expressed ligands on self-tissues and act to dampen unwanted immune activation. In theory, a T cell which has escaped central tolerance, with a potentially autoreactive TCR, will be prevented from causing harm as it encounters its antigen in the context of healthy self-tissue expressing co-inhibitory ligands and no danger signals. Similar mechanisms control the response of innate immune cells to other inflammatory signals.

### Immune checkpoint receptors as targets in cancer

In recent years it has become clear that cancers can co-opt these immune checkpoint pathways to evade the immune system, and therapeutic antibodies that block these receptors can take the brakes off the anti-tumor immune response, with astonishing results. An antibody blocking the receptor CTLA4 was the first to show efficacy in treating malignant melanoma (1), followed by antibodies blocking PD1 or its ligand PDL1 (2). These new immunotherapies, known as checkpoint inhibitors, have revolutionized the treatment of metastatic melanoma. They offer a subset of patients a durable remission from a disease that was previously invariably terminal. Since these initial trials checkpoint inhibitors have gone on to show efficacy in a wide range of other cancers (3) and whilst the list of indications for CTLA4 and PD1 blockade is growing, other immune inhibitory receptors are being investigated as potential targets in cancer therapy (4).

One of the limitations of checkpoint inhibitors has been the new genre of side effect they have led to, referred to as immune related adverse events (IRAEs). Treated patients can develop a wide range of autoimmune phenomena affecting almost any organ, including the gut, skin, pituitary, thyroid, lung, liver, joints, kidneys, pancreas, or haematopoietic system (5). These adverse events highlight the importance of immune checkpoint receptors in maintaining self-tolerance and raise the question of to what extent defects in these pathways could be contributing to spontaneous autoimmune disease.

### Immune checkpoint defects in autoimmunity

In both humans and mice immune checkpoint receptors have been shown to play a crucial role in preserving peripheral tolerance. CTLA4 knock out mice develop massive lymphoproliferation and die of multiorgan tissue destruction early in life (6), whilst human patients with heterozygous loss of function mutations in CTLA4 also develop widespread immune dysregulation (7). PD1 knockout mice on a BALB/c background develop autoimmune cardiomyopathy (8) whilst on a C57BL/6 background they develop a late onset lupus-like disease (9). In humans regulatory polymorphisms in the *PDCD1* gene are associated with susceptibility to a variety of autoimmune conditions including systemic lupus erythematosus (10), atopy and rheumatoid arthritis (11, 12), and progression in multiple sclerosis (MS) (13). It is in fact possible that the therapeutic benefit of interferon-beta in MS may be due to it upregulating PDL1 expression on myeloid cells (14). Furthermore, autoantibodies against PDL1 have been found in patients with rheumatoid arthritis and correlate with disease activity (15).

In addition to PD1 and CTLA4 there are numerous other immune checkpoint receptors that have been shown to have important immune regulatory function. B- and T-lymphocyte attenuator (BTLA) knock-out mice gradually develop multi-organ inflammatory infiltrates and a hepatitis-like disease (16), whilst a gene polymorphism in humans is associated with rheumatoid arthritis (17). Mice lacking T cell Immunoreceptor with Ig and ITIM domains (TIGIT) do not develop spontaneous autoimmunity but have increased susceptibility to experimental autoimmune encephalitis (EAE) (18). Similarly, mice without Lymphocyte-activation gene 3 (LAG3) do not develop spontaneous disease but have accelerated diabetes onset when bred onto a NOD background. Polymorphisms of the T cell immunoglobulin and mucin domain 3 (TIM-3) receptor in humans have been associated with MS (19), rheumatoid arthritis (20) and ankylosing spondylitis (21).

### Rationale for targeting immune checkpoints in autoimmunity

The association of immune checkpoint receptors with autoimmunity in humans and the autoimmune phenomena seen when these receptors are knocked out in experimental mice or blocked therapeutically in patients all offer evidence of the crucial role these pathways play in regulating immune responses. It also raises the possibility that inducing signaling through these receptors could switch off detrimental immune responses and drive the immune system back toward a state of tolerance after control has been lost in autoimmune disease. This idea has been explored for a range of different targets and in multiple mouse models of autoimmunity (summarized in Table 1). Below we will review attempts that have been made to date to create agonistic compounds capable of delivering inhibitory signals to T cells through checkpoint receptors. Such inhibitory agonists, if they could be translated into human disease, would comprise a new, broadly useful class of immunosuppressive drug (see Table 2: Summary of key points).

**Table 1:** Checkpoint agonists that have shown efficacy in treating mouse models of autoimmunity.

**Target receptor**	**Agonist compound**	**Mouse disease model**	**References**
PD-1	mPDL1-mIgG2a^mut^ Fc fusion^*^	CIA CIA DSS/T cell colitis	(22) (23) (24)
	hPDL1-hIgG4 Fc fusion	Islet transplant	(25)
	PDL1 transfected dendritic cells	EAE	(26)
BTLA	mHVEM-mIgG1 Fc fusion	GVHD	(27)
	mHVEM-hIgG1 Fc fusion	Cardiac allograft	(28)
	Hamster IgG antibody (clone 6A6)	GVHD	(29)
	Rat IgG antibody (clone Byk-1)	GVHD	(30)
TIGIT	Armenian hamster IgG antibody (4D4)	EAE	(31)
TIM-3	Galectin 9	EAE, Cardiac allograft, Skin allograft, CIA	(32) (33) (34) (35)
CD200 Receptor	mCD200-mIgG2a^mut^	CIA CIA Rat islet xenograft	(36) (37) (38)
	mCD200-mIgG2a	EAE	(39)
	Rat IgG1 antibody (clone OX110)	CIA Influenza infection	(40) (41)
	Rat IgG1 antibody (clone DX109)	Autoimmune uveoretinitis	(42)
	DNA aptamers	Skin graft	(43)
CD200R/ TGFβR	CD200—TGFβ fusion protein	Skin graft	(44)
VISTA	Armenian hamster antibody (MH5A)	GVHD	(45)
	Mouse IgG1 antibody (mam82)	Concanavalin A hepatitis	(46)
Unknown	Pentameric VISTA-COMP fusion protein	Skin allograft	(47)

* *mIgG2a^mut^ contains the mutations E318A, K320A, K322A to inactivate the C1q binding site and L235E to reduce FcγR1 binding*.

**Table 2:** Summary of key points.

Checkpoint receptors deliver inhibitory signals to immune cells to prevent inappropriate or excessive activation The absence or blockade of these receptors leads to autoimmunity Conversely, inducing signaling through these pathways could help to switch off unwanted immune responses for the treatment of autoimmune disease Agonist antibodies, Fc-fusion proteins and other novel compounds that trigger these receptors have demonstrated promise in treating animal models of autoimmunity, but this has not yet been translated to human disease The epitope position, along with an ability to bind to Fc receptors, and to cause receptor aggregation, all play a role in determining the potency of an agonist compound Better understanding the mechanisms by which agonists induce signaling could direct the design of more effective therapeutic agents

## Inhibitory agonists targeting immune checkpoints in mouse models of autoimmunity

### Agonistic agents based on natural ligands

One therapeutic approach to induce signaling through co-inhibitory receptors has been to make use of their naturally occurring ligands. Ligand expression is normally confined to specific tissues and cell types, but by systemic administration of recombinantly produced ligand it is possible to induce inhibitory signaling through a receptor in tissues where this pathway is not normally functioning, thereby supplementing the body's natural tolerance checkpoints. The simplest application of this is demonstrated by the TIM-3 ligand Galectin-9 which, when administered as a soluble protein to mice, ameliorated EAE (35), prolonged skin and cardiac allograft survival (33, 34), and reduced inflammation in collagen induced arthritis (CIA) (35). However, the promiscuous nature of galectins, binding to sugars on multiple different glycoproteins, makes it difficult to definitively attribute these effects to TIM-3 signaling rather than the manipulation of another galectin-9 binding partner (48).

Galectin-9 is a rare example of a ligand that has been successfully employed as a standalone protein. A more widespread approach is to express the ligand as an Fc fusion, linked to the hinge and constant domains (CH2 and CH3) of an immunoglobulin heavy chain. The potential advantages of an added Fc region include easier protein expression and purification, and extended serum half-life. Furthermore, expression as an Fc fusion dimerises the ligand, turning relatively low receptor affinities into substantially higher avidities, as well as enabling receptor crosslinking. The ability of the Fc portion to be captured by Fc receptors on antigen presenting cells also effectively turns the ligand into an immobilized cell surface receptor rather than a soluble protein.

Several attempts have been made to target the potent inhibitory receptor PD1 with Fc fusions. A construct comprising murine PDL1 with mIgG2a^mut^ Fc (mutated to inactivate the C1q and FcγR1 binding sites) dampened collagen-specific T cell responses and improved clinical scores in CIA (22, 23). An adenovirus vector expressing the same construct ameliorated dextran sodium sulfate-induced experimental colitis, whilst the recombinant form reduced the severity of T-cell induced colitis (24). A human PDL1 hIgG4 fusion protein delayed rejection of islet cell transplants in mice but only when used in conjunction with CD40L blockade (25).

The CD200 receptor (CD200R), predominantly expressed on myeloid cells, has also had success as a target for ligand-Fc inhibitory agonists. A mCD200-mIgG2a^mut^ fusion protein prevented CIA when given alongside collagen immunization (36) and significantly delayed rejection of rat-to-mouse islet xenografts (38). Separately, mCD200-mIgG2a was able to reduce disease severity in established arthritis (37) and, via suppression of microglia and astrocyte activity, attenuated disease in EAE (39). The latter two studies did not specify whether the Fc construct used contained the same mutations removing high affinity FcR and complement binding, so cytotoxic depletion of CD200R1 expressing cells may have been a contributing factor.

Fc fusions of HVEM, the ligand for the inhibitory receptor BTLA have also displayed promise as immunosuppressants. Mouse or human HVEM-IgG1 fusion proteins inhibited T cell responses *in vitro*, but only when crosslinked by a secondary antibody or when high molecular weight aggregates were present (49). *In vivo*, mHVEM-hIgG1 prolonged survival of cardiac allografts when used in combination with cyclosporine (28) and mHVEM-mIgG1 ameliorated a model of graft vs. host disease (GVHD) (27). Conversely mHVEM-hIgG1 exacerbated CIA (50) which may have been due to inducing inflammatory signaling through the activating co-receptor LIGHT which also binds to HVEM. As highlighted here many inhibitory receptors such as BTLA act in paired systems, sharing their ligands with activating receptors (Table 3), which presents a challenge to utilizing the natural ligands as immunosuppressive agents. For example, CD80-Fc and CD86-Fc fusion proteins which may be expected to have an inhibitory effect on T cells via CTLA4 signaling, in fact have a net activating effect due to also binding CD28, and have been shown to enhance anti-tumor immune responses (51).

**Table 3:** Selected immune checkpoint receptors alongside their ligands and paired receptors.

**Checkpoint receptor**	**Ligands**	**Paired** **receptors (sharing the same ligand)**
CTLA4 (cytotoxic T lymphocyte associated protein 4)	CD80, CD86	Activating: CD28
PD1 (Programmed cell death protein 1)	PDL1, PDL2	–
BTLA (B- and T-Lymphocyte attenuator)	HVEM (Herpesvirus entry mediator)	Activating: LIGHT, LTα Inhibitory: CD160
TIGIT (T cell Immunoreceptor with Ig and ITIM domains)	CD155, CD112	Activating: CD226 Inhibitory: CD96
CD200 Receptor (CD200R1)	CD200	Activating: CD200R2-5 (mice only, not expressed in humans)
TIM-3 (T cell immunoglobulin and mucin domain 3)	Galectin 9, HMGB1, Phosphatidylserine, CEACAM-1	Numerous
LAG-3 (Lymphocyte-activation gene 3)	MHC Class II	Activating: T cell receptor, CD4
VISTA (V-domain Ig suppressor of T cell activation)	Unknown (VISTA may also serve as a co-inhibitory ligand for an, as yet, unidentified receptor)	–

### Agonist antibodies

In contrast to natural ligands, therapeutic antibodies can be produced which have specificity for only the inhibitory partner in paired receptor systems, avoiding the risk of inducing counterproductive signaling through activating receptors. Antibodies can also be selected with many-fold higher affinity for their cognate receptor than the affinity of the endogenous receptor-ligand interaction. Furthermore, the significant precedent for monoclonal antibodies to be used as therapeutics in humans, could mean that translation to the clinic will face fewer challenges than would be encountered by novel Fc-fusions or other innovative constructs.

It was demonstrated long ago in the context of the activating co-stimulatory receptor CD28, that antibodies could substitute for natural ligands, and in fact could deliver a far more potent signal (52). This was confirmed in an unfortunate way in the clinical trial of the CD28 superagonist antibody TGN1412 in which widespread T cell activation caused a cytokine storm in the participating healthy volunteers (53). Conversely, agonistic antibodies against inhibitory receptors have shown promise in mouse models of autoimmunity, although there are currently very few registered clinical trials of agonists against these targets in humans.

Krieg et al. screened eight rat anti-mouse BTLA antibodies and found one with significant agonistic activity, which was able to inhibit CD4 T cell activation when immobilized, even if delivered 24 h after the initial anti-CD3 activation signal (54). Separately, a hamster IgG targeting BTLA abrogated disease in a model of GVHD in wildtype but not BTLA^−/−^ C57BL/6 mice (29). Of note, this antibody had previously been shown to block binding of the natural ligand HVEM (55), but as it was capable of ameliorating disease even in HVEM^−/−^ mice, and was shown to be non-depleting, Albring et al concluded the effect must be due to direct signaling through BTLA.

An IgG1 rat anti-mouse CD200R1 antibody (OX110) reduced disease severity in overtly arthritic mice (40) and alleviated influenza-induced illness by dampening excessive innate cell activation (41). Another rat IgG1 antibody against mouse CD200R1 (DX109) suppressed macrophage activation and prevented tissue damage in experimental autoimmune uveitis (42). *In vitro* DX109 was able to inhibit degranulation of CD200R1 overexpressing mast cells, whilst a rat anti-human CD200R antibody (DX183) suppressed primary human mast cells (56).

Targeting the receptor VISTA (PD-1H), an Armenian hamster IgG prevented GVHD by tolerising effector T cells and selectively promoting regulatory T cell (Treg) expansion (57), whilst a mIgG1 VISTA agonist antibody suppressed acute inflammation in a model of Concanavalin-A induced hepatitis (46). Dixon et al recently described a mIgG1 antibody targeting the receptor TIGIT which suppressed T cell responses to immunization with myelin oligodendrocyte glycoprotein (MOG) peptide and modulated disease severity in EAE (31).

The success of CTLA4 and PD1 as targets of checkpoint blockade in cancer highlights these two receptors as particularly crucial regulators of tolerance. So it is conspicuous that no successful attempts to utilize agonist antibodies against these receptors in treating autoimmunity has been published. In the case of CTLA4 this may add weight to the suggestion that the receptor does not have an important intrinsic signaling capability but instead acts predominantly by sequestering the ligands CD86 and CD80, preventing their interaction with CD28 (58). This is supported by the clinical success of the CTLA4-Fc fusion protein Abatacept, which is used in the treatment of rheumatoid arthritis amongst other indications (59). Rather than acting as an agonist, like the Fc-fusion proteins described above, Abatacept acts as a blocking agent, binding to CD80 and CD86 on antigen presenting cells and preventing their co-stimulatory interaction with CD28 on T cells. The fact that soluble CTLA4-Fc is a potent immunosuppressive and can compensate for CTLA4 haploinsufficiency (60) suggests that competition with CD28 for ligand binding is the predominant mode of action of this inhibitory receptor. However, there is also substantial evidence for an intrinsic signaling function of CTLA4 and a membrane bound single chain antibody (ScFv) recognizing CTLA-4 has been reported as having a T cell suppressive effect if expressed on the same cell as the TCR antigen, suggesting that it may be possible to develop agonist antibodies against this receptor (61, 62).

The immune checkpoint PD1 does have a potent intrinsic signaling function so the reason for a lack of successful agonists targeting this receptor is unclear. There are reports of a PD1 antibody ameliorating autoimmunity in a lupus-like disease model in mice (63–65) but, as this antibody had previously been shown to act as a PD1 blocking agent, the authors attributed the effect to either cytotoxic depletion of PD1 expressing cells or enhanced suppressive activity of Tregs following PD1 blockade. Based on the efficacy of PDL1/Fc fusion proteins in murine models of autoimmunity described above, whether antibody agonists targeting PD1 can be developed is an area that certainly merits wider exploration.

### Novel approaches to checkpoint agonism

Aside from agonist antibodies and ligand/Fc fusions a variety of other constructs have been employed to induce immunosuppressive signaling through inhibitory receptors. Cheung et al. exploited the cytomegalovirus protein UL144, which binds to BTLA and is presumably used by the virus as an immune evasion strategy, and showed that immobilized UL144-Fc more potently suppressed CD4 T cells *in vitro* than HVEM-Fc. Šedý et al. studied the structure of UL144 to guide their design of a mutated HVEM-Fc protein capable of binding BTLA with 10 fold higher affinity than wildtype HVEM and with no binding to the receptors LIGHT or CD160. *In vitro* this construct regulated B, T, and NK cell cytokine production (66). There are numerous other viral proteins that have evolved to mimic inhibitory ligands, which presents an opportunity to further explore these compounds as therapeutic agents and once again highlights the potential merits of exploiting signaling through inhibitory receptors to switch off unwanted immune responses.

In another innovative approach to inhibitory agonism, a bivalent construct of CD200Fc linked to TGF-β1 displayed more potent T cell suppression *in vitro* than either protein alone, and prolonged survival of allogeneic skin grafts *in vivo* (44). In mixed leucocyte reactions (MLRs), binding to CD200R on antigen presenting cells and TGF-β receptor on responder T cells was shown to be necessary for maximal suppressive effect. Separately, Prodeus et al. developed short single-stranded DNA aptamers with binding specificity for CD200R1 and demonstrated that they were capable of suppressing T cell function in MLRs, whilst a PEGylated DNA aptamer prolonged skin graft survival with equal efficacy to CD200-Fc (43). Finally, a pentameric construct of VISTA fused to the pentamerization domain from cartilage oligomeric matrix protein (COMP) prolonged skin allograft survival and rescued mice from acute concanavalin-A-induced hepatitis, although, assuming that this construct functions as an inhibitory agonist, it is not known what receptor it is targeting (47).

The idea of overexpressing an inhibitory ligand on dendritic cells to produce a tolerogenic cell that can be used as a therapeutic agent has also been investigated. Dendritic cells transfected with both PDL1 and MOG peptide and injected intraperitoneally were able to induce tolerance and reduce severity of MOG-induced EAE (26). Similarly, splenocytes from Balb/c mice primed with allogeneic dendritic cells overexpressing PDL1 and loaded with GAD65 had impaired responses when subsequently stimulated with the same antigens *ex vivo* (67). However, whether transfected dendritic cells could ever be translated into an acceptable therapeutic for use in human autoimmune disease is uncertain.

## Rational development of checkpoint agonists

### Defining the necessary characteristics for a checkpoint agonist

For a compound to act as an immune checkpoint agonist it not only has to bind to the receptor but must also be capable of delivering a signal through it. Very little has been done to establish the criteria that determine this function. Despite the development of the numerous agonists described above there is still little clarity as to what characteristics are necessary in an agent to confer upon it this agonistic ability.

#### Agonists to TNFR family receptors

In the context of activating TNFR family immune cell receptors, such as CD40, it has been demonstrated that antibody agonism results from receptor aggregation, which in turn is dependent on capture of the antibody, via its Fc portion, by a scaffold of FcγRIIB on the surface of adjacent cells (68). As such, agonist activity can be augmented by increasing affinity for FcγRIIB (69). Furthermore, FcγRIIB independent agonism can be conferred by an isoform of human IgG2 in which the CH1 domain is linked via a disulfide bond to the hinge, which holds the antibody in a more compact and rigid structure and presumably aids tighter packing or more efficient aggregation of bound receptors (70).

#### Mechanism of triggering of checkpoint receptors

However, it is important to remember that TNFR family receptors fall into a different family from the inhibitory immune receptors we have discussed here, with different signaling mechanisms and, presumably, different attributes necessary for agents acting as agonists. TNFR family receptors are normally engaged by multivalent ligands and signal after receptor trimerization leads to the recruitment of downstream adapter proteins. Immune checkpoint receptors on the other hand predominantly fall into a category of receptors that have been referred to as non-catalytic tyrosine phosphorylated receptors or NTRs (71). These receptors have tyrosine containing motifs in their cytoplasmic tail that become phosphorylated by extrinsic kinases following ligand binding, which in turn leads to recruitment of SH2 domain-containing downstream signaling proteins or adapters. Understanding the mechanism by which ligand engagement leads to phosphorylation of these intracellular motifs (referred to as receptor triggering) is clearly crucial to understanding how artificial agonists might operate. There are several different, but not necessarily mutually exclusive, models for how this process can occur based on the aggregation, conformational change or segregation of membrane proteins (71).

**Figure 1:**
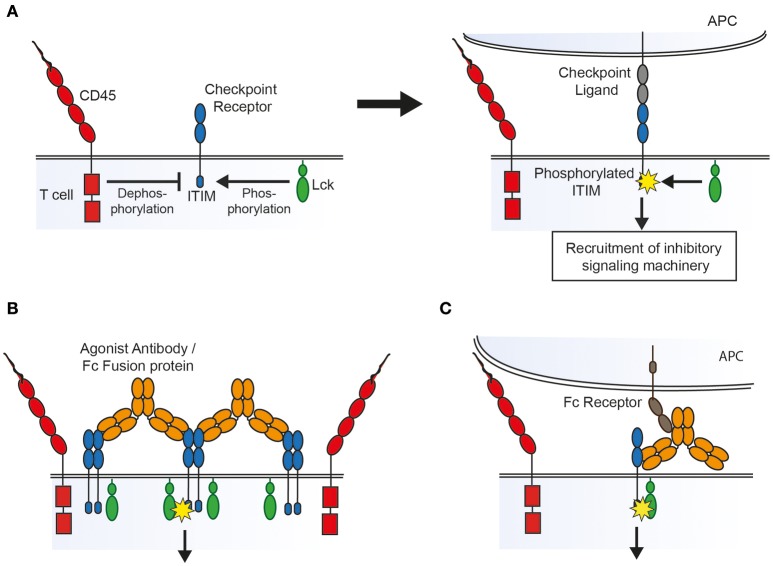
Possible mechanisms of action of agonist agents, based on the kinetic-segregation model of receptor signaling. **(A)** The kinetic-segregation model. (Left) Checkpoint receptors contain intracellular motifs such as the ITIM which are phosphorylated by small membrane associated kinases (e.g., Lck) but rapidly dephosphorylated by abundant bulky phosphatases (e.g., CD45), with no net signaling. (Right) When the receptor encounters its ligand on an apposing cell the balance of kinase and phosphatase activity is tipped in favor of kinases, for example by steric exclusion of phosphatases from the contact zone, resulting in net phosphorylation of the ITIM and subsequent recruitment of signaling machinery which inhibits cellular activation. **(B)** Triggering by aggregation. An agonist compound may cause receptor triggering by densely clustering kinase-associated receptors so that bulky phosphatases are again sterically excluded. **(C)** Triggering by an Fc receptor immobilized compound. An agonist that binds to Fc receptors on an apposing cell could lead to triggering by holding the receptor in a close contact zone that phosphatases cannot enter.

Receptor aggregation models dictate that ligand binding leads to clustering of receptors that, at rest, are loosely associated with intracellular kinases, leading to cross-phosphorylation of tyrosine containing motifs on adjacent receptors. Conformational change models require ligand binding to lead to structural changes in the receptor which either expose previously buried signaling motifs or allow subsequent receptor aggregation. In contrast, the kinetic-segregation model proposes that binding to ligand on an apposing cell holds the receptor in a close contact formed between the two cell surfaces from which bulky receptor-type phosphatases are excluded, which in turn leads to net phosphorylation by kinases that are not excluded because they are associated with the inner leaflet of the membrane (Figure 1A) (72).

#### Aggregation of checkpoint receptors

As with TNFR family receptor agonists there is some evidence that aggregation plays a role in the action of checkpoint agonists. Many of the agonists described above have been shown to inhibit immune cells more potently *in vitro* when crosslinked by a secondary antibody. Also, most of the agonists described above are at least dimeric, and therefore capable of bringing together two of their cognate receptors (and clustering multiple receptors if their cognate receptors themselves oligomerise). The mere fact that soluble natural ligands function as agonists only when dimerised in the form of an Fc fusion protein lends some support to the idea that aggregation is important. Galectin 9, whilst not dimeric, has 2 separate carbohydrate recognition domains capable of binding TIM-3, and can cluster receptors into glycoconjugates which may either induce signaling directly or alter the half-life of the receptor on the cell surface (73). For the checkpoint receptor CD200R it has been shown that agonist antibody isotype is also key, with the compact isoform of human IgG2, which aids receptor clustering, serving to enhance agonism, as seen for TNFR family receptors (74). As inhibitory receptors are thought generally to associate with phosphatases rather than kinases it might seem paradoxical that aggregating them would lead to receptor phosphorylation. However, along the lines of the kinetic-segregation model described above, it may be that clustering receptors into a tightly packed group creates an area of densely occupied membrane from which bulky phosphatases are excluded, allowing for net phosphorylation of signaling motifs by smaller membrane-associated or intracellular kinases (Figure 1B).

#### Requirement for Fc receptor binding

There is also evidence that Fc receptor binding is important to the action of agonists against NTRs. The superagonistic activity of the antibody TGN1412 which targeted the costimulatory receptor CD28 was found to depend on binding to FcγRIIB *in vitro* (75). Similarly, agonistic antibodies targeting the murine inhibitory receptor FcγRIIB, which is itself an NTR, require that both their variable and Fc portions are able to bind Fc receptors (76). As in the case of TNFR agonists, the requirement for Fc receptor binding may be because it aids receptor clustering. Alternatively, if the kinetic-segregation mechanism of receptor triggering plays a role, then Fc receptor binding would be expected to be necessary as the agonistic agent would need to be immobilized on an opposing surface (such as an FcR expressing cell) in order to create the close contact zone which excludes phosphatases (Figure 1C).

Of course the requirement for Fc receptor binding also raises the possibility that the immunosuppressive effects of checkpoint “agonists” could be due to inadvertent depletion of checkpoint expressing effector T cells. Very few of the publications cited above, which showed inhibitory effects on the overall immune response, used assays (such as Phospho-Flow or western blotting) to look at the downstream signaling of these receptors and confirm that the agents were truly agonistic. Furthermore, few convincingly demonstrated that there was no cytotoxic depletion of effector T cells. Recent data suggesting that the immune enhancing effects of CTLA4 “blocking” antibodies may in fact be due to the FcR-dependent depletion of T-regs (77) highlights that we should remain open minded about the potential mechanism of action of novel therapeutics.

#### Epitope position

A number of studies suggest that epitope position may influence the agonistic activity of monoclonal antibodies. From a panel of anti-BTLA antibodies Zhang et al. demonstrated that all those with agonistic effects mapped to the same epitope whilst non-agonists bound elsewhere (78). Interestingly, it does not seem to matter if the antibody competes with binding of the natural ligand. Agonists targeting TIGIT (31) and BTLA (29) were both shown to inhibit ligand binding and to be capable of treating disease in models of autoimmunity.

Clues to how epitope position may be influencing agonist activity come from studies of the activating receptor CD28. It has been demonstrated that superagonist antibodies targeting this receptor bind to a shared epitope on a laterally exposed loop of the receptor (79) and that this results in a relatively compact structure with the antibody lying close to and parallel to the membrane (80). This means that when the antibody Fc portion is immobilized by Fc receptors on an opposing cell, the receptor may be held in a very close contact between the two membranes. In the kinetic-segregation mechanism of receptor triggering, a narrower contact zone would more effectively exclude phosphatases to initiate signaling. Of note, Evans et al. have shown that superagonistic and non-superagonist antibodies are equally capable of binding CD28 bivalently, and so a differential ability to cause receptor aggregation is unlikely to account for the difference in activity. Furthermore, they saw insufficient structural rearrangements of CD28 following antibody binding for a conformational change-based mechanism to readily explain triggering. The idea that the epitope influences agonism because of the resulting width of the gap between cells is supported by the fact that cytotoxic antibodies used clinically tend to target molecules with small extracellular domains such as CD20 (rituximab) and CD52 (campath-1). These antibodies mediate antibody dependent cell mediated cytotoxicity (ADCC) by binding to activatory Fc receptors, which fall into the same NTR family, so may also be dependent on the small dimensions of the interaction. Chimeric antigen receptors (CARs) and bispecific T cell engagers (BiTEs), which both act as artificial NTRs, targeting larger proteins such as CD22 and FcRH5 are most effective if they bind a membrane proximal epitope (81, 82).

#### Co-localization of inhibitory and activating signals

Finally, there is evidence to suggest that the function of inhibitory agonists depends on the co-incidence of inhibitory and activating signals within the cell. For example, a LAG-3 agonistic antibody was able to inhibit T cell proliferation *in vitro* only when co-crosslinked with the TCR by a secondary antibody (83). In addition, the effect of CD200R agonists on mast cell degranulation is enhanced by co-crosslinking to the FcεR (56), and BTLA agonists are effective *in vitro* only when presented alongside the activating anti-CD3 antibody (78). This fits with a mechanism of signaling in which inhibitory receptors recruit phosphatases capable of dephosphorylating the signaling motifs of neighboring activatory receptors. In the context of therapeutic inhibitory agonists, it suggests that a useful agonist will need to be capable of accessing the immune synapse where T cell activation is occurring.

### Choice of mouse model

A variety of murine autoimmune models have been used to assess the effects of inhibitory agonists *in vivo*. It may be that disease associations seen with human checkpoint polymorphisms can give clues to which tissues are more dependent on these pathways for maintaining tolerance and guide the selection of disease model. Similarly, the prevalence of different autoimmune manifestations in checkpoint blockade-treated patients may aid this process. For example, involvement of the pituitary is a relatively common adverse event with CTLA4 blockade, occurring in 10% of patients, but is very rare following PD1 blockade suggesting that different pathways can have tissue specific importance (84). Whether this is due to tissue specific differences in ligand expression or some other factor is unclear but as new blocking antibodies targeting different checkpoint receptors make their way into clinical trials, more information about the organ specific relevance of different pathways will become available. The specific diseases seen in knockout mice may also direct the selection of disease models.

However, it does not necessarily follow that the parts of the body worst affected by blockade or absence of a particular checkpoint receptor would serve to benefit most from agonist agents targeting this receptor. It may be that tissues which develop disease following checkpoint blockade are those where these inhibitory pathways are constitutively active, and that other tissues which don't normally have functional signaling through these receptors are more prone to spontaneous autoimmunity, and more likely to benefit from artificially-induced inhibitory signaling.

There are also many difficulties in extrapolating findings from mouse models back to human disease. For example, whilst PD1 blockade in man leads most often to autoimmunity affecting the gut, liver, and skin, in PD1 knockout mice autoimmune manifestations include cardiomyopathy in BALB/c mice and lupus like disease in C57BL/6 mice, suggesting that knock out models do not always phenocopy the effect of blocking antibodies in man. As seen with the CD28 superagonist TGN1412, not even primate studies can always accurately predict the effects of therapeutic antibodies in man (85).

### Rationale for agonist combinations

With checkpoint inhibitors in cancer we have seen that combination blockade of both CTLA4 and PD1 is superior to either alone (86), and similarly we may expect that combining agonists against multiple pathways may enhance immunosuppression. The choice of combinations to use may be guided by the effects seen in double knockout mice. For example, LAG-3 or VISTA deficiency alone does not lead to spontaneous autoimmunity, but does exacerbate disease in the absence of PD1 (87, 88). Further clues toward synergistic combinations may be gathered from more in-depth understanding of the different downstream signaling pathways of these unique and non-redundant receptors, as well as the expression pattern on different cells of the immune system (89).

### Risk of cancer

The success of checkpoint blockade has highlighted the key role the immune system can play in cancer surveillance and raises the issue of whether inhibitory agonists could aid developing tumors to escape the immune response. There is no suggestion so far from animal models that inhibitory agonists might increase cancer risk, but the timescale of such experiments might be insufficient for this to be clear and longer-term observation of treated mice could be useful. The long experience to date with other clinically used immunosuppressives, however, has been that the increased cancer risk is likely very small, if it is increased at all, and outweighed by the clinical benefit of immune suppression in the context of debilitating autoimmune disease.

## Conclusion

Previous reviews that have discussed immune cell co-receptors as potential targets in autoimmunity have focused primarily on agents that block the action of activating receptors (90–92). Here instead we have concentrated on attempts that have been made to enhance the signaling of inhibitory receptors. Whilst this approach has displayed significant promise in animal models of autoimmunity there is a need for more thorough investigation of the mechanisms underlying artificial agonism of checkpoint receptors, to guide more rational design of the most potent agonists. This, alongside reasoned approaches to selecting the most appropriate combinations of agents and the best models to test them in, could help to unveil the true potential of this previously untapped class of therapeutic antibodies.

## Author contributions

All authors listed have made a substantial, direct and intellectual contribution to the work, and approved it for publication.

### Conflict of interest statement

The authors declare that the research was conducted in the absence of any commercial or financial relationships that could be construed as a potential conflict of interest.

## References

[1] Hodi FS O'Day SJ McDermott DF Weber RW Sosman JA Haanen JB. Improved survival with ipilimumab in patients with metastatic melanoma. N Engl J Med. (2010) 363:711–23. 10.1056/NEJMoa1003466 20525992 PMC3549297

[2] Robert C Long GV Brady B Dutriaux C Maio M Mortier L. Nivolumab in previously untreated melanoma without BRAF mutation. N Engl J Med. (2015) 372:320–30. 10.1056/NEJMoa1412082 25399552

[3] Gong J Chehrazi-Raffle A Reddi S Salgia R. Development of PD-1 and PD-L1 inhibitors as a form of cancer immunotherapy: a comprehensive review of registration trials and future considerations. J Immunother Cancer (2018) 6:8. 10.1186/s40425-018-0316-z 29357948 PMC5778665

[4] Le Mercier I Lines JL Noelle RJ. Beyond CTLA-4 and PD-1, the generation Z of negative checkpoint regulators. Front Immunol. (2015) 6:418. 10.3389/fimmu.2015.00418 26347741 PMC4544156

[5] Michot JM Bigenwald C Champiat S Collins M Carbonnel F Postel-Vinay S. Immune-related adverse events with immune checkpoint blockade: a comprehensive review. Eur J Cancer (2016) 54:139–48. 10.1016/j.ejca.2015.11.016 26765102

[6] Tivol EA Borriello F Schweitzer AN Lynch WP Bluestone JA Sharpe AH. Loss of CTLA-4 leads to massive lymphoproliferation and fatal multiorgan tissue destruction, revealing a critical negative regulatory role of CTLA-4. Immunity (1995) 3:541–7. 10.1016/1074-7613(95)90125-6 7584144

[7] Kuehn HS Ouyang W Lo B Deenick EK Niemela JE Avery DT. Immune dysregulation in human subjects with heterozygous germline mutations in CTLA4. Science (2014) 345:1623–7. 10.1126/science.1255904 25213377 PMC4371526

[8] Nishimura H Okazaki T Tanaka Y Nakatani K Hara M Matsumori A. Autoimmune dilated cardiomyopathy in PD-1 receptor-deficient mice. Science (2001) 291:319–22. 10.1126/science.291.5502.319 11209085

[9] Nishimura H Nose M Hiai H Minato N Honjo T. Development of lupus-like autoimmune diseases by disruption of the PD-1 gene encoding an ITIM motif-carrying immunoreceptor. Immunity (1999) 11:141–51. 10.1016/S1074-7613(00)80089-8 10485649

[10] Prokunina L Castillejo-López C Oberg F Gunnarsson I Berg L Magnusson V. A regulatory polymorphism in PDCD1 is associated with susceptibility to systemic lupus erythematosus in humans. Nat Genet. (2002) 32:666–9. 10.1038/ng1020 12402038

[11] James ES Harney S Wordsworth BP Cookson WO Davis SJ Moffatt MF. PDCD1: a tissue-specific susceptibility locus for inherited inflammatory disorders. Genes Immun. (2005) 6:430–7. 10.1038/sj.gene.6364223 15959535

[12] Lee YH Bae SC Kim JH Song GG. Meta-analysis of genetic polymorphisms in programmed cell death 1. Associations with rheumatoid arthritis, ankylosing spondylitis, and type 1 diabetes susceptibility. Z Rheumatol. (2015) 74:230–9. 10.1007/s00393-014-1415-y 24942602

[13] Kroner A Mehling M Hemmer B Rieckmann P Toyka KV Mäurer M. A PD-1 polymorphism is associated with disease progression in multiple sclerosis. Ann Neurol. (2005) 58:50–7. 10.1002/ana.20514 15912506

[14] Schreiner B Mitsdoerffer M Kieseier BC Chen L Hartung HP Weller M. Interferon-beta enhances monocyte and dendritic cell expression of B7-H1 (PD-L1), a strong inhibitor of autologous T-cell activation: relevance for the immune modulatory effect in multiple sclerosis. J Neuroimmunol. (2004) 155:172–82. 10.1016/j.jneuroim.2004.06.013 15342209

[15] Dong H Strome SE Matteson EL Moder KG Flies DB Zhu G. Costimulating aberrant T cell responses by B7-H1 autoantibodies in rheumatoid arthritis. J Clin Invest. (2003) 111:363–70. 10.1172/JCI16015 12569162 PMC151851

[16] Oya Y Watanabe N Owada T Oki M Hirose K Suto A. Development of autoimmune hepatitis-like disease and production of autoantibodies to nuclear antigens in mice lacking B and T lymphocyte attenuator. Arthritis Rheum. (2008) 58:2498–510. 10.1002/art.23674 18668554 PMC2782777

[17] Oki M Watanabe N Owada T Oya Y Ikeda K Saito Y. A functional polymorphism in B and T lymphocyte attenuator is associated with susceptibility to rheumatoid arthritis. Clin Dev Immunol. (2011) 2011:305656. 10.1155/2011/305656 21403914 PMC3049324

[18] Joller N Hafler JP Brynedal B Kassam N Spoerl S Levin SD. Cutting edge: TIGIT has T cell-intrinsic inhibitory functions. J Immunol. (2011) 186:1338–42. 10.4049/jimmunol.1003081 21199897 PMC3128994

[19] Yaghoobi E Abedian S Babani O Izad M. TIM-3 Rs10515746 (A/C) and Rs10053538 (C/A) Gene polymorphisms and risk of multiple sclerosis. Iran J Public Health (2016) 45:644–49. 27398337 PMC4935708

[20] Chae SC Park YR Shim SC Yoon KS Chung HT. The polymorphisms of Th1 cell surface gene Tim-3 are associated in a Korean population with rheumatoid arthritis. Immunol Lett. (2004) 95:91–5. 10.1016/j.imlet.2004.06.008 15325803

[21] Wang M Ji B Wang J Cheng X Zhou Q Zhou J. Tim-3 polymorphism downregulates gene expression and is involved in the susceptibility to ankylosing spondylitis. DNA Cell Biol. (2014) 33:723–8. 10.1089/dna.2014.2456 24905803

[22] Raptopoulou AP Bertsias G Makrygiannakis D Verginis P Kritikos I Tzardi M. The programmed death 1/programmed death ligand 1 inhibitory pathway is up-regulated in rheumatoid synovium and regulates peripheral T cell responses in human and murine arthritis. Arthritis Rheum. (2010). 62:1870–80. 10.1002/art.27500 20506224

[23] Wang G Hu P Yang J Shen G Wu X. The effects of PDL-Ig on collagen-induced arthritis. Rheumatol Int. (2011) 31:513–9. 10.1007/s00296-009-1249-0 20035333

[24] Song MY Hong CP Park SJ Kim JH Yang BG Park Y. Protective effects of Fc-fused PD-L1 on two different animal models of colitis. Gut (2015) 64:260–71. 10.1136/gutjnl-2014-307311 24902766

[25] Gao W Demirci G Strom TB Li XC. Stimulating PD-1-negative signals concurrent with blocking CD154 co-stimulation induces long-term islet allograft survival. Transplantation (2003) 76:994–9. 10.1097/01.TP.0000085010.39567.FB 14508368

[26] Hirata S Senju S Matsuyoshi H Fukuma D Uemura Y Nishimura Y Prevention of experimental autoimmune encephalomyelitis by transfer of embryonic stem cell-derived dendritic cells expressing myelin oligodendrocyte glycoprotein peptide along with TRAIL or programmed death-1 ligand. J Immunol. (2005) 174:1888–97. 10.4049/jimmunol.174.4.1888 15699115

[27] Brown GR Lee EL El-Hayek J Kintner K Luck C. IL-12-independent LIGHT signaling enhances MHC class II disparate CD4^+^ T cell alloproliferation, IFN-γ responses, and intestinal graft-versus-host disease. J Immunol. (2005) 174:4688–95. 10.4049/jimmunol.174.8.4688 15814693

[28] Ye Q Fraser CC Gao W Wang L Busfield SJ Wang C. Modulation of LIGHT-HVEM costimulation prolongs cardiac allograft survival. J Exp Med. (2002) 195:795–800. 10.1084/jem.20012088 11901205 PMC2193745

[29] Albring JC Sandau MM Rapaport AS Edelson BT Satpathy A Mashayekhi M. Targeting of B and T lymphocyte associated (BTLA) prevents graft-versus-host disease without global immunosuppression. J Exp Med. (2010) 207:2551–59. 10.1084/jem.20102017 21078889 PMC2989771

[30] Sakoda Y Park JJ Zhao Y Kuramasu A Geng D Liu Y. Dichotomous regulation of GVHD through bidirectional functions of the BTLA-HVEM pathway. Blood (2011) 117:2506–14. 10.1182/blood-2010-08-301325 21220749 PMC3062413

[31] Dixon KO Schorer M Nevin J Etminan Y Amoozgar Z Kondo T. Functional Anti-TIGIT antibodies regulate development of autoimmunity and antitumor immunity. J Immunol. (2018) 200:3000–7 10.4049/jimmunol.1700407 29500245 PMC5893394

[32] Zhu C Anderson AC Schubart A Xiong H Imitola J Khourys SJ. The Tim-3 ligand galectin-9 negatively regulates T helper type 1 immunity. Nat Immunol. (2005) 6:1245–52. 10.1038/ni1271 16286920

[33] He W Fang Z Wang F Wu K Xu Y Zhou H. Galectin-9 significantly prolongs the survival of fully mismatched cardiac allografts in mice. Transplantation (2009) 88:782–90. 10.1097/TP.0b013e3181b47f25 19920777

[34] Wang F He W Zhou H Yuan J Wu K Xu L. The Tim-3 ligand galectin-9 negatively regulates CD8+ alloreactive T cell and prolongs survival of skin graft. Cell Immunol. (2007) 250:68–74. 10.1016/j.cellimm.2008.01.006 18353298

[35] Seki M Oomizu S Sakata KM Sakata A Arikawa T Watanabe K. Galectin-9 suppresses the generation of Th17, promotes the induction of regulatory T cells, and regulates experimental autoimmune arthritis. Clin Immunol. (2008) 127:78–88. 10.1016/j.clim.2008.01.006 18282810

[36] Gorczynski RM Chen Z Yu K Hu J. CD200 immunoadhesin suppresses collagen-induced arthritis in mice. Clin Immunol. (2001) 101:328–34. 10.1006/clim.2001.5117 11726225

[37] Simelyte E Criado G Essex D Uger RA Feldmann M Williams RO. CD200-Fc, a novel antiarthritic biologic agent that targets proinflammatory cytokine expression in the joints of mice with collagen-induced arthritis. Arthritis Rheum. (2008) 58:1038–43. 10.1002/art.23378 18383359

[38] Gorczynski RM Hu J Chen Z Kai Y Lei J. A CD200FC immunoadhesin prolongs rat islet xenograft survival in mice. Transplantation (2002) 73:1948–53. 10.1097/00007890-200206270-00018 12131694

[39] Liu Y Bando Y Vargas-Lowy D Elyaman W Khoury SJ Huang T. CD200R1 agonist attenuates mechanisms of chronic disease in a murine model of multiple sclerosis. J Neurosci. (2010) 30:2025–38. 10.1523/JNEUROSCI.4272-09.2010 20147531 PMC2837938

[40] Gorczynski RM Chen Z Lee L Yu K Hu J. Anti-CD200R ameliorates collagen-induced arthritis in mice. Clin Immunol. (2002) 104:256–64. 10.1006/clim.2002.5232 12217336

[41] Snelgrove RJ Goulding J Didierlaurent AM Lyonga D Vekaria S Edwards L. A critical function for CD200 in lung immune homeostasis and the severity of influenza infection. Nat Immunol. (2008) 9:1074–83. 10.1038/ni.1637 18660812

[42] Copland DA Calder CJ Raveney BJ Nicholson LB Phillips J Cherwinski H. Monoclonal antibody-mediated CD200 receptor signaling suppresses macrophage\ activation and tissue damage in experimental autoimmune uveoretinitis. Am J Pathol. (2007) 171:580–88. 10.2353/ajpath.2007.070272 17600119 PMC1934542

[43] Prodeus A Cydzik M Abdul-Wahid A Huang E Khatri I Gorczynski R. Agonistic CD200R1 DNA aptamers are potent immunosuppressants that prolong allogeneic skin graft survival. Mol Ther Nucleic Acids (2014) 3:e190. 10.1038/mtna.2014.41 25158092 PMC4221601

[44] Gorczynski RM Chen Z Shivagnahnam S Taseva A Wong K Yu K. Potent immunosuppression by a bivalent molecule binding to CD200R and TGF-betaR. Transplantation (2010) 90:150–9. 10.1097/TP.0b013e3181e2d6a1 20548263

[45] Flies DB Wang S Xu H Chen L. Cutting edge: a monoclonal antibody specific for the programmed death-1 homolog prevents graft-versus-host disease in mouse models. J Immunol. (2011) 187:1537–41. 10.4049/jimmunol.1100660 21768399 PMC3150865

[46] Flies DB Han X Higuchi T Zheng L Sun J Ye J. Coinhibitory receptor PD-1H preferentially suppresses CD4^+^ T cell-mediated immunity. J Clin Invest. (2014) 124:1966–75. 10.1172/JCI74589 24743150 PMC4001557

[47] Prodeus A Abdul-Wahid A Sparkes A Fischer NW Cydzik M Chiang N. VISTA.COMP - an engineered checkpoint receptor agonist that potently suppresses T cell-mediated immune responses. JCI Insight (2017) 2:e94308. 10.1172/jci.insight.94308 28931757 PMC5621893

[48] Cooper DN Barondes SH. God must love galectins; he made so many of them. Glycobiology (1999) 9:979–84. 10.1093/glycob/9.10.979 10521533

[49] Gonzalez LC Loyet KM Calemine-Fenaux J Chauhan V Wranik B Ouyang W. A coreceptor interaction between the CD28 and TNF receptor family members B and T\ lymphocyte attenuator and herpesvirus entry mediator. Proc Natl Acad Sci USA. (2005) 102:1116–21. 10.1073/pnas.0409071102 15647361 PMC544343

[50] Pierer M Schulz A Rossol M Kendzia E Kyburz D Haentzschel H. Herpesvirus entry mediator-Ig treatment during immunization aggravates rheumatoid arthritis in the collagen-induced arthritis model. J Immunol. (2009) 182:3139–45. 10.4049/jimmunol.0713715 19234211

[51] Sturmhoefel K Lee K Gray GS Thomas J Zollner R O'Toole M. Potent activity of soluble B7-IgG fusion proteins in therapy of established tumors and as vaccine adjuvant. Cancer Res. (1999) 59:4964–72. 10519410

[52] Tacke M Hanke G Hanke T Hünig T. CD28-mediated induction of proliferation in resting T cells *in vitro* and *in vivo* without engagement of the T cell receptor: evidence for functionally distinct forms of CD28. Eur J Immunol. (1997) 27:239–47. 10.1002/eji.1830270136 9022025

[53] Suntharalingam G Perry MR Ward S Brett SJ Castello-Cortes A Brunner MD. Cytokine storm in a phase 1 trial of the anti-CD28 monoclonal antibody TGN1412. N Engl J Med. (2006). 355:1018–28. 10.1056/NEJMoa063842 16908486

[54] Krieg C Han P Stone R Goularte OD Kaye J. Functional analysis of B and T lymphocyte attenuator engagement on CD4^+^ and CD8^+^ T cells. J Immunol. (2005) 175:6420–7. 10.4049/jimmunol.175.10.6420 16272294

[55] Hurchla MA Sedy JR Gavrieli M Gavrielli M Drake CG Murphy TL. B and T lymphocyte attenuator exhibits structural and expression polymorphisms and is highly Induced in anergic CD4^+^ T cells. J Immunol. (2005) 174:3377–85. 10.4049/jimmunol.174.6.3377 15749870

[56] Cherwinski HM Murphy CA Joyce BL Bigler ME Song YS Zurawski SM. The CD200 receptor is a novel and potent regulator of murine and human mast cell \ function. J Immunol. (2005) 174:1348–56. 10.4049/jimmunol.174.3.1348 15661892

[57] Flies DB Higuchi T Chen L. Mechanistic assessment of PD-1H coinhibitory receptor-induced T cell tolerance to allogeneic antigens. J Immunol. (2015) 194:5294–304. 10.4049/jimmunol.1402648 25917101 PMC4433880

[58] Walker LS Sansom DM. Confusing signals: recent progress in CTLA-4 biology. Trends Immunol. (2015) 36:63–70. 10.1016/j.it.2014.12.001 25582039 PMC4323153

[59] Moreland L Bate G Kirkpatrick P Abatacept. Nat Rev Drug Discov. (2006) 5:185–6. 10.1038/nrd1989 16557658

[60] Lee S Moon JS Lee CR Kim HE Baek SM Hwang S. Abatacept alleviates severe autoimmune symptoms in a patient carrying a de novo variant in CTLA-4. J Allergy Clin Immunol. (2016) 137:327–30. 10.1016/j.jaci.2015.08.036 26478010

[61] Griffin MD Hong DK Holman PO Lee KM Whitters MJ O'Herrin SM. Blockade of T cell activation using a surface-linked single-chain antibody to CTLA-4 (CD152). J Immunol. (2000) 164:4433–42. 10.4049/jimmunol.164.9.4433 10779742

[62] Fife BT Griffin MD Abbas AK Locksley RM Bluestone JA. Inhibition of T cell activation and autoimmune diabetes using a B cell surface-linked CTLA-4 agonist. J Clin Invest. (2006) 116:2252–61. 10.1172/JCI27856 16886063 PMC1523399

[63] Kasagi S Kawano S Okazaki T Honjo T Morinobu A Hatachi S. Anti-programmed cell death 1 antibody reduces CD4^+^PD-1^+^ T cells and relieves the lupus-like nephritis of NZB/W F1 mice. J Immunol. (2010) 184:2337–47. 10.4049/jimmunol.0901652 20139271

[64] Wong M La Cava A Singh RP Hahn BH. Blockade of programmed death-1 in young (New Zealand black x New Zealand white)F1 mice promotes the activity of suppressive CD8^+^ T cells that protect from lupus-like disease. J Immunol. (2010) 185:6563–71. 10.4049/jimmunol.0903401 21041733

[65] Wong M La Cava A Hahn BH. Blockade of programmed death-1 in young (New Zealand Black x New Zealand White)F1 mice promotes the suppressive capacity of CD4^+^ regulatory T cells protecting from lupus-like disease. J Immunol. (2013) 190:5402–10. 10.4049/jimmunol.1202382 23636058 PMC3700538

[66] Šedý JR Balmert MO Ware BC Smith W Nemčovičova I Norris PS. A herpesvirus entry mediator mutein with selective agonist action for the inhibitory receptor B and T lymphocyte attenuator. J Biol Chem. (2017) 292:21060–70. 10.1074/jbc.M117.813295 29061848 PMC5743079

[67] He FR Zhu HF Huang H Dai YD Shen X Wang M. Programmed death-1 ligands-transfected dendritic cells loaded with glutamic acid decarboxylase 65 (GAD65) inhibit both the alloresponse and the GAD65-reactive lymphocyte response. Clin Exp Immunol. (2008) 151:86–93. 10.1111/j.1365-2249.2007.03546.x 18005363 PMC2276913

[68] Li F Ravetch JV. A general requirement for FcγRIIB co-engagement of agonistic anti-TNFR antibodies. Cell Cycle (2012) 11:3343–44. 10.4161/cc.21842 22918247 PMC3466534

[69] Dahan R Barnhart BC Li F Yamniuk AP Korman AJ Ravetch JV. Therapeutic activity of agonistic, human anti-CD40 monoclonal antibodies requires selective FcγR engagement. Cancer Cell (2016) 29:820–31. 10.1016/j.ccell.2016.05.001 27265505 PMC4975533

[70] White AL Chan HT French RR Willoughby J Mockridge CI Roghanian A. Conformation of the human immunoglobulin G2 hinge imparts superagonistic properties to immunostimulatory anticancer antibodies. Cancer Cell (2015) 27:138–48. 10.1016/j.ccell.2014.11.001 25500122 PMC4297290

[71] Dushek O Goyette J van der Merwe PA. Non-catalytic tyrosine-phosphorylated receptors. Immunol Rev. (2012) 250:258–76. 10.1111/imr.12008 23046135

[72] Davis SJ van der Merwe PA. The kinetic-segregation model: TCR triggering and beyond. Nat Immunol. (2006) 7:803–809. 10.1038/ni1369 16855606

[73] Belardi B O'Donoghue GP Smith AW Groves JT Bertozzi CR. Investigating cell surface galectin-mediated cross-linking on glycoengineered cells. J Am Chem Soc. (2012) 134:9549–52. 10.1021/ja301694s 22540968 PMC3374418

[74] Grujic O Stevens J Chou RY Weiszmann JV Sekirov L Thomson C. Impact of antibody subclass and disulfide isoform differences on the biological activity of CD200R and βklotho agonist antibodies. Biochem Biophys Res Commun. (2017) 486:985–91. 10.1016/j.bbrc.2017.03.145 28363871

[75] Bartholomaeus P Semmler LY Bukur T Boisguerin V Römer PS Tabares P. Cell contact-dependent priming and Fc interaction with CD32^+^ immune cells contribute to the TGN1412-triggered cytokine response. J Immunol. (2014) 192:2091–2098. 10.4049/jimmunol.1302461 24470499

[76] Williams EL Tutt AL French RR Chan HT Lau B Penfold CA. Development and characterisation of monoclonal antibodies specific for the murine inhibitory FcγRIIB (CD32B). Eur J Immunol. (2012) 42:2109–2120. 10.1002/eji.201142302 22760702

[77] Du X Tang F Liu M Su J Zhang Y Wu W. A reappraisal of CTLA-4 checkpoint blockade in cancer immunotherapy. Cell Res. (2018) 28:416–32. 10.1038/s41422-018-0011-0 29472691 PMC5939050

[78] Zhang M Howard K Winters A Steavenson S Anderson S Smelt S Monoclonal antibodies to B and T lymphocyte attenuator (BTLA) have no effect on *in vitro* B cell proliferation and act to inhibit *in vitro* T cell proliferation when presented in a cis, but not trans, format relative to the activating stimulus. Clin Exp Immunol. (2011) 163:77–87. 10.1111/j.1365-2249.2010.04259.x 21078085 PMC3010914

[79] Lühder F Huang Y Dennehy KM Guntermann C Müller I Winkler E. Topological requirements and signaling properties of T cell-activating, anti-CD28 antibody superagonists. J Exp Med. (2003) 197:955–66. 10.1084/jem.20021024 12707299 PMC2193880

[80] Evans EJ Esnouf RM Manso-Sancho R Gilbert RJ James JR Yu C. Crystal structure of a soluble CD28-Fab complex. Nat Immunol. (2005) 6:271–9. 10.1038/ni1170 15696168

[81] James SE Greenberg PD Jensen MC Lin Y Wang J Till BG. Antigen sensitivity of CD22-specific chimeric TCR is modulated by target epitope distance from the cell membrane. J Immunol. (2008) 180:7028–38. 10.4049/jimmunol.180.10.7028 18453625 PMC2585549

[82] Li J Stagg NJ Johnston J Harris MJ Menzies SA DiCara D. Membrane-proximal epitope facilitates efficient T cell synapse formation by anti-FcRH5/CD3 and is a requirement for myeloma cell killing. Cancer Cell (2017) 31:383–95. 10.1016/j.ccell.2017.02.001 28262555 PMC5357723

[83] Hannier S Tournier M Bismuth G Triebel F. CD3/TCR complex-associated lymphocyte activation gene-3 molecules inhibit CD3/TCR signaling. J Immunol. (1998) 161:4058–65. 9780176

[84] Faje A. Immunotherapy and hypophysitis: clinical presentation, treatment, and biologic insights. Pituitary (2016) 19:82–92. 10.1007/s11102-015-0671-4 26186958

[85] Pallardy M Hünig T. Primate testing of TGN1412: right target, wrong cell. Br J Pharmacol. (2010) 161:509–11. 10.1111/j.1476-5381.2010.00925.x 20880391 PMC2990150

[86] Wolchok JD Kluger H Callahan MK Postow MA Rizvi NA Lesokhin AM. Nivolumab plus ipilimumab in advanced melanoma. N Engl J Med. (2013) 369:122–33. 10.1056/NEJMoa1302369 23724867 PMC5698004

[87] Okazaki T Okazaki IM Wang J Sugiura D Nakaki F Yoshida T. PD-1 and LAG-3 inhibitory co-receptors act synergistically to prevent autoimmunity in mice. J Exp Med. (2011) 208:395–407. 10.1084/jem.20100466 21300912 PMC3039848

[88] Liu J Yuan Y Chen W Putra J Suriawinata AA Schenk AD. Immune-checkpoint proteins VISTA and PD-1 nonredundantly regulate murine T-cell responses. Proc Natl Acad Sci USA. (2015) 112:6682–7. 10.1073/pnas.1420370112 25964334 PMC4450438

[89] Nirschl CJ Drake CG. Molecular pathways: coexpression of immune checkpoint molecules: signaling pathways and implications for cancer immunotherapy. Clin Cancer Res. (2013) 19:4917–24. 10.1158/1078-0432.CCR-12-1972 23868869 PMC4005613

[90] Ford ML Adams AB Pearson TC. Targeting co-stimulatory pathways: transplantation and autoimmunity. Nat Rev Nephrol. (2014) 10:14–24. 10.1038/nrneph.2013.183 24100403 PMC4365450

[91] Murakami N Riella LV. Co-inhibitory pathways and their importance in immune regulation. Transplantation (2014) 98:3–14. 10.1097/TP.0000000000000169 24978034

[92] Zhang Q Vignali DA. Co-stimulatory and co-inhibitory pathways in autoimmunity. Immunity (2016) 44:1034–51. 10.1016/j.immuni.2016.04.017 27192568 PMC4873959

